# Nova fails to appreciate the value of plant‐based meat and dairy alternatives in the diet

**DOI:** 10.1111/1750-3841.70039

**Published:** 2025-02-10

**Authors:** Mark Messina, Virgina Messina

**Affiliations:** ^1^ Soy Nutrition Institute Global Jefferson City Missouri USA; ^2^ Nutrition Matters, Inc. Pittsfield Massachusetts USA

**Keywords:** Nova, plant‐based meat alternatives, plant milks, soy, ultra‐processed foods

## Abstract

Processed foods play an important role in achieving both food and nutrition security. However, in recent years, there has been increased concern about the health effects of food processing, in large part because of the emergence of the Nova food classification system. Nova classifies all foods into one of four groups purportedly based entirely on the extent to which they have been processed. Recommendations to limit intake of ultra‐processed foods (UPF) (group 4) are based primarily on observational studies showing that their intake is associated with a range of adverse outcomes. Nearly all plant milks and the entire new generation of plant‐based meat alternatives (PBMAs), which are made using concentrated sources of plant protein, are classified as UPFs. This classification may deter the public from consuming and health professionals from recommending these products even though they represent a convenient way to increase plant protein intake in high‐income countries, which is recommended by health authorities. However, although total UPF intake is associated with adverse health outcomes, this is not the case for many subcategories of UPFs. Furthermore, in many instances, clinical research shows that PBMAs and plant milks have beneficial effects relative to their animal‐based counterparts (Group 1). Collectively, the evidence leads to two conclusions. First, PBMAs represent a viable approach for lowering the dietary animal to plant protein ratio. Second, Nova paints with too broad a brush and is insufficiently nuanced to serve as a public guide for food purchasing decisions and may distract consumers from focusing on the importance of nutrient content.

## 1 INTRODUCTION

For both health (Al‐Shaar et al., 2020; Naghshi et al., 2020; Song et al., 2016) and environmental (Gibbs & Cappuccio, 2022; Willett et al., 2019) reasons, global health authorities are increasingly calling for high‐income countries to shift toward more plant‐forward diets and to replace some animal protein with plant protein. Currently, in the United States, approximately two thirds of protein is derived from animal sources and one third from plants, which is similar to the ratios in other high‐income countries (Halkjaer et al., 2009; Pasiakos et al., 2015). In low‐income countries, that ratio is reversed (Grigg, 1995; Our World in Data, 2024). Although there is widespread agreement about the need for a shift toward more plant protein in high‐income countries, specific recommendations vary. For example, the EAT‐Lancet Commission recommends obtaining most protein from plant sources (Willett et al., 2019), whereas the Dutch Food‐Based Dietary Guidelines recommend a 1:1 ratio (Grasso et al., 2021). The latter recommendation aligns with those from the 2020‐2025 US Dietary Guidelines Advisory Committee which call for greater emphasis on consuming sources of plant protein and with Canada's food guide, which recommends choosing plant‐based protein foods more often as an alternative to animal protein (Government of Canada, 2024).

Pulses, which include dry beans, such as dry broad beans, dry peas, chickpeas, cow peas, pigeon peas, and lentils, are a desirable option for increasing plant protein intake because of their affordability (Drewnowski & Rehm, 2013), small environmental footprint (Harwatt et al., 2017), and proposed health benefits (Becerra‐Tomas et al., 2019; Mullins & Arjmandi, 2021). However, they account for only 6% of total protein intake globally, and in the United States, dry beans account for only 2% and 6% of total protein and plant protein intake, respectively (Shan et al., 2019). Furthermore, several lines of evidence indicate that the intake of pulses consumed in the traditional manner is unlikely to substantially increase in the foreseeable future (ANSES, 2019; Askew, 2020; Grasso & Jaworska, 2020; Melendrez‐Ruiz et al., 2019; Ryan & Deci, 2000). Multiple observations illustrate the difficulty of making substantial dietary change (Tao et al., 2022).

Plant‐based meat and dairy alternatives may provide a more convenient and appealing means of increasing plant protein intake because they share similar culinary roles in the diet as the products they are intended to replace. The new generation of plant‐based meat alternatives (PBMAs) more closely mimics the orosensory properties (taste and texture) of meat than earlier iterations and has made greater inroads among mainstream consumers than previous versions of these foods (Gonzales et al., 2023). Research shows that for PBMAs to successfully replace meat, they need to emulate the taste, texture, visual appearance, and preparation of meat (Michel et al., 2021; Szenderak et al., 2022). Although these products are popular with vegetarians, they are designed to appeal to a much broader demographic of people who want to reduce meat intake or incorporate new foods into their diet (Szejda & Matti, 2020). In general, the new PBMAs consist of a concentrated source of one or more proteins usually classified as an isolate (≥90% protein) or concentrate (≥65% protein) (Codex General Standard for Soy Protein Products, Codex Standard 175–1989, 1989), in addition to fat, binding agents, colorants, and flavoring agents.

Despite their seeming advantages, concerns about the role of both plant‐based meat and dairy alternatives in a healthy diet have arisen, especially regarding the former. These concerns fall into two general categories. One is that many of the potentially beneficial bioactives found in whole plant foods are absent to varying degrees in PBMAs (Edge & Garrett, 2020; Flint et al., 2023; Gonzales et al., 2023; Hu et al., 2019; Ketelings et al., 2023; Macdiarmid, 2021; Toh et al., 2022; Tso & Forde, 2021). Another is that the processing involved in the manufacture of these products may lead to adverse health effects (Vellinga et al., 2024). For example, thermal processing used to reduce microbial contamination may induce the formation of carcinogens in PBMAs, just as it does in meat products. Moreover, processing can affect the bioavailability, digestibility, and nutritional characteristics of foods and ingredients (Bogueva & McClements, 2023). These concerns have been heightened by the emergence of the Nova food classification system (Haneberg et al., 2024; Monteiro et al., 2016) and the categorization of PBMAs as ultra‐processed.

In this article, we propose that Nova is an inadequate means of evaluating the healthfulness of foods in general and plant‐based meat and dairy alternatives in particular and is insufficiently nuanced to serve as a guide for consumer food purchasing decisions. Despite their classification as ultra‐processed foods (UPFs), we maintain that plant‐based meat and dairy alternatives can contribute to a healthy diet while playing a role in increasing plant protein intake. Further, the acceptance of Nova by health agencies may potentially cause harm to public health since many foods designated as UPFs may in fact play important roles in healthy diets. Nova may also serve to distract consumers from focusing on the importance of nutrient content.

## 2 FOOD PROCESSING

As noted, concerns have been raised about the potential harmful effects of food processing related to the loss of bioactive compounds and production of harmful compounds. However, there are benefits associated with food processing including food safety (e.g., chemical, physical, and biological risks and hazards), prolongation of shelf‐life, increased nutrient content through fortification, improved nutrient availability, increased convenience and affordability, reduction in post‐harvesting losses, and preservation of desirable sensory qualities (flavor, texture, aroma, and appearance) (Michel et al., 2024; van Het Hof et al., 2000).

According to the American Society for Nutrition, processed foods play a role in achieving both food security (ensuring that sufficient food is available) and nutrition security (ensuring that food quality meets human nutrient needs) (Weaver et al., 2014). Processed foods make food preparation and nutritious meals more accessible to people with limited financial resources, physical and cognitive limitations, inadequate access to food preparation facilities and equipment, limited access to fresh foods, or a lack of time or skills required for cooking.

Nevertheless, US (and global) consumers have expressed increased concern about the possible adverse health effects of modern food processing (Meijer et al., 2021), although most (70%) do not fully understand or cannot explain what a processed food is (International Food Information Council, 2024). Evidence suggests that consumers use perceived degree of processing as a cue for perceived healthfulness (Bolhuis et al., 2022). Hassig et al. (2023) reported a strong negative correlation (*r* = −0.96) between mean perceived processing and perceived healthiness of the food. Interest in “clean eating” has placed an emphasis on unprocessed or whole foods, products with short ingredient lists and familiar ingredients, along with efforts to avoid artificial colors or flavors, additives and preservatives, and genetically modified foods. Many consumers are seeking organic or environment‐friendly/sustainable farming methods (Ambwani et al., 2020).

**FIGURE 1:**
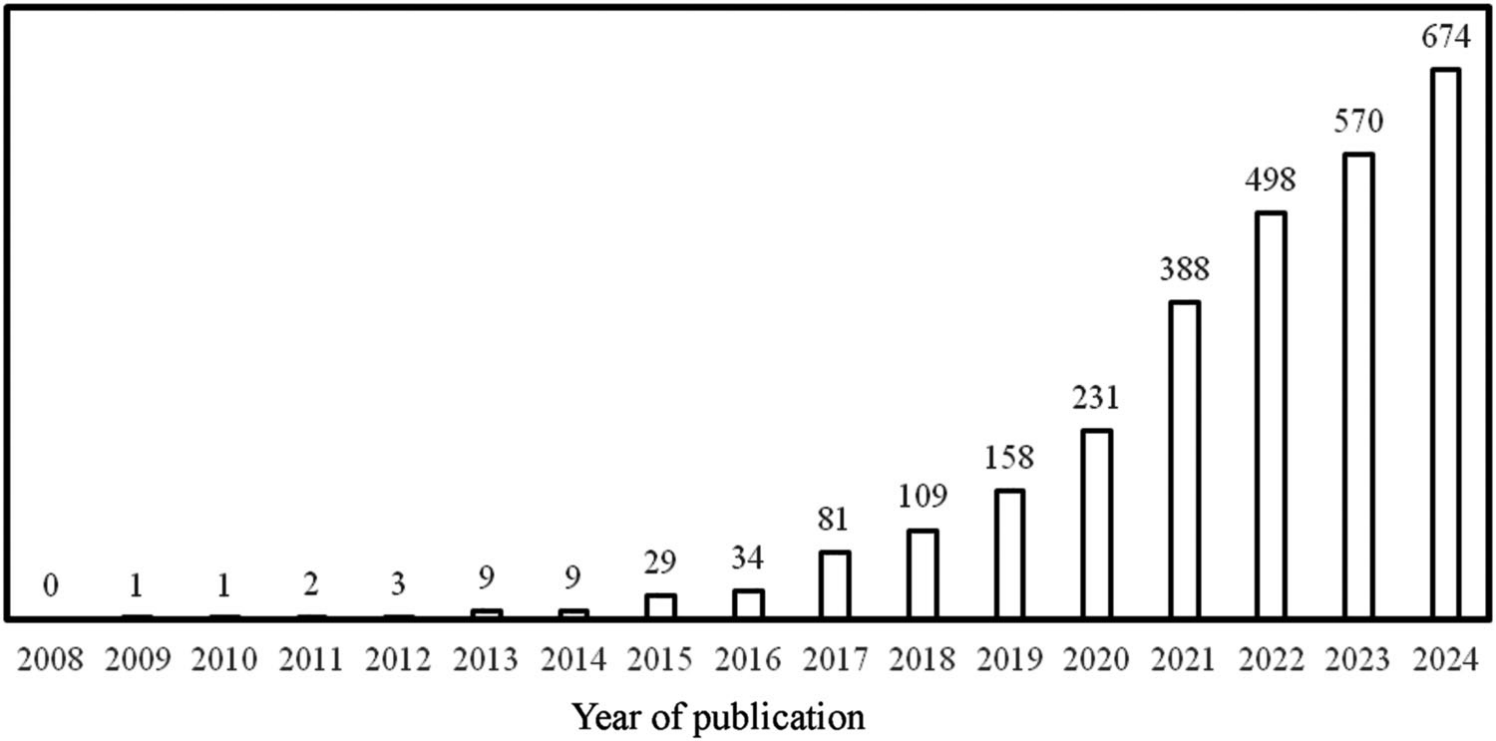
Annual number of articles indexed in PubMed (for years 2008–2024) that include the terms ultra‐processed or ultraprocessed.

The health effects of food processing have received much recent attention from the research community. In 2024, nearly 700 manuscripts that refer to UPFs were indexed in PubMed, an approximate 3‐fold increase from 2020 and a 20‐fold increase from 2016 (Figure 1). The heightened consumer and scientific interest in food processing can be attributed at least partially to the emergence of the Nova food classification system and its concept of UPFs (Haneberg et al., 2024; Monteiro et al., 2016). As evidence of the impact of Nova on consumer perception, a recent US survey revealed that 41% of adults actively avoid UPFs (IFIC, 2024).

Processing can impact the health effects of food even without changing nutrient content. For example, the thickness of an oat flake and disruption in the structural integrity of the oat kernel influence the glycemic response to this food even when nutrient or fiber content is unaltered (Musa‐Veloso et al., 2021). The disruption of the food matrix can also impact nutrient absorption. For example, the percentage of fat absorbed from whole peanuts is lower than from peanut butter (Levine & Silvis, 1980). Satiety may also be affected by processing as fruit in solid form has greater satiety than pureed fruit or juice (Flood‐Obbagy & Rolls, 2009).

## 3 THE NOVA SYSTEM AND ULTRA‐PROCESSED FOODS

### 3.1 Origins and perspective

The term “ultra‐processed” first appeared in a 1982 *Esquire* article, by Laurence Shames (Logan et al., 2024). In his article, *The Universal Salesman*, Shames called out the marketing executives who, he said, peddled “ultra‐processed foodstuffs whose labels read like a chem text.” Seven years later, Leo D. Galland referred to the 1970s–1980s as the “era of ultra‐processed foods” (Logan et al., 2024). However, the term UPF became part of the nutrition lexicon only with the development of Nova in 2009 by Brazilian scientists (Haneberg et al., 2024; Martinez‐Steele et al., 2023; Monteiro et al., 2016).

In 2009, Monteiro (2009) proposed classifying all foods and drinks into one of three groups: minimally processed foods (Group 1), substances extracted from whole foods (Group 2), and UPFs (Group 3). UPFs were defined as those made from Group 2 substances to which “ … either no or relatively small amounts of minimally processed foods from group 1 are added, plus salt and other preservatives, and often also cosmetic additives—flavours and colours.” To prevent disease and promote well‐being, Monteiro recommended avoiding or at least minimizing intake of UPFs irrespective of nutrient content. Monteiro (2010) and Monteiro et al. (2010) expanded upon this system for classifying all foods into one of three groups in papers published in 2010.

Not until 2018 was the Nova food classification system officially proposed, and at that time, a change was made to this system so that foods were divided into four groups instead of three (Monteiro et al., 2018). Nova is based on the nature, extent, and purpose of food processing and places foodstuffs into one of four groups: unprocessed/minimally processed (Group 1); processed culinary ingredients (Group 2); processed foods (Group 3); and UPFs (Group 4) (Table 1). UPFs are “formulations of industrial sources of dietary energy and nutrients, particularly unhealthy types of fat, starches, free sugars, and salt, plus additives including those designed to intensify sensory impact. They typically contain little or even no intact food.” Much of the impetus for the creation of Nova can be attributed to the global epidemic of obesity and chronic disease, which correlates with a shift away from foods that make up the bulk of traditional diets to UPFs produced by large transnational corporations (Monteiro, 2010).

**TABLE 1:** Nova food group definitions as per extent and purpose of industrial food processes.

Group	Definition	Examples
1 (Unprocessed or minimally processed)	Unprocessed foods: edible parts of plants (fruits, seeds, leaves, stems, roots, tubers) or of animals (muscle, offals, eggs, milk), and also fungi, algae and water, after separation from nature. Minimally processed foods: unprocessed foods altered by industrial processes such as removal of inedible or unwanted parts, drying, crushing, grinding, fractioning, roasting, toasting, boiling, pasteurization, refrigeration, freezing, placing in containers, vacuum packaging, nonalcoholic fermentation, and other methods that largely preserve the food matrix and do not add salt, sugar, oils or fats or other food substances to the original food. The main aim of these processes is to extend the life of unprocessed foods, enabling their storage for longer use, and, often, to make their preparation easier or more diverse. Additives are usually not necessary and only exceptionally found in minimally processed foods	Fresh, squeezed, chilled, frozen, or dried fruits and leafy and root vegetables; grains such as brown, parboiled or white rice, corn cob or kernel, wheat berry or grain; legumes such as beans, lentils and chickpeas; starchy roots and tubers such as potatoes, sweet potatoes and cassava; fungi such as fresh or dried mushrooms and yeast; meat, poultry, fish and seafood, whole or in the form of steak fillets and other cuts, fresh or chilled or frozen; eggs; fresh or pasteurized milk; fresh or pasteurized plain yogurt; fresh or pasteurized fruit or vegetable juices (with no added sugar, sweeteners, or flavors); grits, flakes or flour made from corn, wheat, oats or cassava; raw or toasted tree and ground nuts and other oily seeds (with no added salt or sugar); herbs and spices used in culinary preparations, such as thyme, oregano, mint, pepper, cloves and cinnamon, whole or powdered, fresh or dried; tea, coffee, and drinking water. Moreover, foods made up of two or more items in this group, such as dried mixed fruits, granola made from cereals, nuts and dried fruits with no added sugar, honey or oil; pasta, couscous and polenta made with flours, flakes or grits and water; and foods with vitamins and minerals added generally to replace nutrients lost during processing, such as wheat or corn flour fortified with iron and folic acid
2 (Processed culinary ingredients)	Substances obtained directly from Group 1 foods or from nature by industrial processes such as pressing, centrifuging, extracting, refining, dewatering, and mining. Processes here aid in the creation of products used in the seasoning and cooking of Group 1 foods and their use in dishes and meals prepared from scratch. Additives are usually not necessary and only exceptionally found in processed culinary ingredients	Vegetable oils crushed from seeds, nuts or fruits (notably olives); butter and lard obtained from milk and pork; sugar and molasses obtained from cane or beet; honey extracted from combs and syrup from maple trees; vinegar; starches extracted from corn and other plants, and salt mined or from seawater. Also, products consisting of two Group 2 items, such as salted butter, and Group 2 items with added vitamins or minerals, such as iodized salt
3 (Processed foods)	Relatively simple industrially manufactured food products made by adding at least one Group 2 ingredient (such as salt, sugar, oil, or fat) to Group 1 foods, using preservation methods such as canning and bottling, and, in the case of breads and cheeses, using nonalcoholic fermentation and boiling or baking. Processes and ingredients here aim to increase the durability of Group 1 foods and make them more enjoyable by modifying or enhancing their sensory qualities. Processed foods often contain additives that prolong product duration, protect original properties, or prevent proliferation of microorganisms (such as preservatives and antioxidants), but not additives with cosmetic functions (see next group)	All canned or bottled vegetables and legumes in brine; salted or sugared nuts and seeds; fruits in syrup; and dried or canned fish. Breads, cheese, pastries, cakes, cookies (biscuits); sweet or savory snacks; cured meats; and ready‐to‐heat products such as burgers, and preprepared pies and pasta and pizza dishes when these products are made exclusively from Group 1 foods and salt, oil, sugar, or other Nova Group 2 ingredients and do not contain classes of additives with cosmetic function
4 (Ultra‐processed foods)	Industrially manufactured food products made up of several ingredients (formulations), including sugar, oils, fats and salt (generally in combination and in higher amounts than in processed foods) and food substances of no or rare culinary use (such as high‐fructose corn syrup, hydrogenated oils, modified starches and protein isolates). Group 1 foods are absent or represent a small proportion of the ingredients in the formulation. Processes enabling the manufacture of ultra‐processed foods include industrial techniques such as extrusion, moulding and pre‐frying; application of additives including those whose function is to make the final product palatable or hyper‐palatable such as flavors, colorants, non‐sugar sweeteners and emulsifiers; and sophisticated packaging, usually with synthetic materials. Processes and ingredients here are designed to create highly profitable (low‐cost ingredients, long shelf‐life, emphatic branding), convenient (ready‐to‐(h)eat or to drink), tasteful alternatives to all other Nova food groups and to freshly prepared dishes and meals. Ultra‐processed foods are operationally distinguishable from processed foods by the presence of food substances of no culinary use (varieties of sugars such as fructose, high‐fructose corn syrup, “fruit juice concentrates,” invert sugar, maltodextrin, dextrose and lactose; modified starches; modified oils such as hydrogenated or interesterified oils; and protein sources such as hydrolyzed proteins, soya protein isolate, gluten, casein, whey protein and “mechanically separated meat”) or of additives with cosmetic functions (flavors, flavor enhancers, colors, emulsifiers, emulsifying salts, sweeteners, thickeners and anti‐foaming, bulking, carbonating, foaming, gelling and glazing agents) in their list of ingredients	All carbonated soft drinks; reconstituted fruit juices and “fruit” drinks; “cocoa” and other dairy drinks, and energy drinks; flavored yogurt; candies (confectionery); margarines; poultry and fish “nuggets” and “sticks,” sausages, hot dogs, luncheon meats and other reconstituted meat products; plant‐based meat substitutes; extruded breakfast “cereals”; powdered “instant” soups, noodles and desserts; infant formulas and “follow‐on” milks; and “health” and “slimming” products such as meal‐replacement shakes and powders. Breads, pastries, cakes, cookies (biscuits); sweet or savory snacks; cured meats; and ready‐to‐heat products such as burgers, and preprepared pies and pasta and pizza dishes when these products are made up of food substances of no culinary use and or contain classes of additives with cosmetic function

*Note*: Alcoholic drinks are not immediately classifiable by Nova. By analogy with the nature of processed and ultra‐processed foods, they may be counted in Group 3 if they are produced by fermentation of Group 1 foods, and in Group 4 if they are produced by fermentation of Group 1 foods and distillation of the resulting alcohol.

*Source*: Adapted with permission from Martinez‐Steele et al. Nature Food (2023), https://doi.org/10.1038/s43016‐023‐00779‐w.

The term UPF was developed within a sociopolitical framework to identify foods manufactured in factories by large companies and does not consider the nutrient profiles of foods. The developers of Nova focus attention on the food industry, not just the food produced by industry. They have compared the techniques used by some manufacturers to increase profits to those used by the tobacco industry (Monteiro, 2010). They also note that transnational companies producing UPFs and drinks are most able to purchase substrates for their products at low prices which allows them to penetrate new markets in lower‐income countries. With large marketing and advertising budgets, they may undercut local industries, drive them out of business, or take them over (Monteiro, 2010). Monteiro et al. (2018) have regularly suggested that the consumption of UPFs negatively affects not just public health but also social structures and food culture.

However, increasingly, the term UPF is less reflective of a sociopolitical context and more commonly used to refer to foods high in fat, sugar, and salt, often considered “junk foods.” Estimates are that UPFs as defined by Nova account for approximately 73% of all foods in the marketplace (Menichetti et al., 2023) and 57%–58% of calories (Juul et al., 2021; Marino et al., 2021; Touvier et al., 2023) consumed in the United States (Touvier et al., 2023). UPF intake among US adults has increased modestly over the last nearly 20 years (Juul et al., 2021), whereas the percentage of energy intake (67%) from UPF is higher in US children than in adults and has increased more over a nearly similar time frame (Wang et al., 2021).

There is considerable concordance between Nutri‐Score, a nutrient profiling model based primarily on nutrient content, and Nova (Sarda et al., 2024). That is, UPFs generally receive poor scores as assessed by Nutri‐Score. The idea of combining Nutri‐Score and Nova for a front‐of‐package label has been explored (Srour et al., 2023). Monteiro et al. (2018) have criticized other food classification systems for placing foods with different effects on health and disease in the same category. However, this criticism may be just as applicable, or even more so, to Nova as some foods categorized as UPFs are found to have beneficial effects on health (Crimarco et al., 2020; Erlich et al., 2024).

In addition, many UPFs are highly rated by other widely used nutrient profiling models such as Health Star Rating (1–5, 5 being the highest score), Nutri‐Score (A–E, A being the highest score), and Food Compass (1–100, foods with scores ≥70 are encouraged). For example, despite being classified as UPFs, Uncle Sam's brand cereal receives scores of 5, A, and 100; fruit salad with nondairy topping receives scores of 4, A, and 80; and beet greens with margarine receives scores of 4, B, and 100 (Mozaffarian et al., 2021).

### 3.2 Influence on food and nutrition policy

The issue of UPF consumption is impacting nutrition perspectives and policy. In 2023, the USDA sponsored a 2‐day workshop on this topic (O'Connor et al., 2023). Moreover, a question addressed by the 2025–2030 US Dietary Guidelines Advisory Committee is “What is the relationship between consumption of dietary patterns with varying amounts of UPFs and growth, body composition, and risk of obesity?” Nova has been embraced by the World Health Organization (WHO) (Monteiro et al., 2019, 2018) and incorporated into the dietary guidelines of several countries (Koios et al., 2022).

There has also been discussion about regulating and taxing UPFs in many regions throughout the world, including the United States, but especially in Mexico and South America (Global Food Research Program. UNC‐Chappel Hill; Valizadeh & Ng, 2024). Taxation of foods and beverages deemed unhealthful is an approach that has precedent and that to some extent has been successful, as taxes have led to shifts in purchasing decisions in some countries (Valizadeh & Ng, 2024), although most focus has been on sugary snacks and sugar‐sweetened beverages (Allcott et al., 2019). In contrast, an analysis by Gangl (2024) did not show taxes on sugar‐sweetened sodas in Hungary and France led to a decrease in soda consumption among school‐aged children.

In addition to taxation, there are calls to regulate UPFs. The European office of the WHO recently called for better labeling of UPFs and for restricting their marketing (Wickramasinghe et al., 2021). In alignment, the authors of a recently published article in Harvard Public Health Magazine called for the US Food and Drug Administration and USDA to regulate and perhaps prohibit the sale of some UPFs (Mande & Delaware, 2023). Warning labels on UPFs have also been proposed (Cotter et al., 2021).

### 3.3 Criticisms of Nova

Nova has served to highlight the importance of consuming unprocessed or minimally processed foods and has led to greater discussion of the health effects of food additives, some of which may not have been thoroughly evaluated and/or not recently evaluated (Pomeranz et al., 2024). However, Nova has been highly criticized for not being functional/usable because UPFs are poorly defined and for not being sufficiently informative because it is redundant and overlaps with other existing nutrient profiling models (Astrup & Monteiro et al., 2022; Braesco et al., 2022; Drewnowski et al., 2020; Gibney et al., 2017; Pereira, 2022; Rodrigues Petrus et al., 2021). Levine and Ubbink (2023) recently opined that Nova demonizes foods because of processing rather than unhealthy formulation and suggests that this can undermine public acceptance of the types of processing that are essential for the development of affordable, sustainable, and nutritious foods that are safe from potential pathogens and that are easily stored and transported.

Furthermore, there are practical difficulties associated with the use of Nova as health professionals with knowledge of ingredients have a difficult time placing foods in their correct Nova categories. To this point, when French food and nutrition specialists were asked to place each of 200 foods into the correct Nova category, only four foods were put into the same Nova category by all the evaluators, and 25% of the foods were placed by different subjects in all four Nova categories (Braesco et al., 2022).

Finally, there is debate as to whether Nova is based more on processing or formulation (Levine & Ubbink, 2023; O'Connor et al., 2024). Although it may be possible to improve the nutrient composition of many UPFs, such as by reducing saturated fat and sugar content, this type of reformulation would not change their designation as UPFs given that even the presence of an emulsifier like lecithin, a common food additive that occurs naturally in many foods and was found to be safe by a recent evaluation by the European Food Safety Authority (Mortensen et al., 2017), qualifies a food as ultra‐processed.

## 4 HEALTH EFFECTS OF UPFS

Observational studies consistently link UPF intake to a range of adverse health outcomes, including certain cancers, diabetes, coronary artery disease, obesity, and depression (Barbaresko et al., 2024; Dai et al., 2024; Lane et al., 2024; Lv et al., 2024; Wang et al., 2024) although the quality of the evidence is often graded as weak (Lane et al., 2024). Although energy density and nutrient content have no bearing on Nova classification, UPFs are often energy dense and nutrient poor (Gupta et al., 2019), and higher UPF consumption has been associated with substantially lower diet quality in several studies (Liu et al., 2022; Torquato et al., 2024). However, in many cases, the associations between UPF intake and adverse health outcomes remain after adjusting for diet quality (Dicken & Batterham, 2021; Fung et al., 2024; Wang et al., 2022).

Very limited clinical work has evaluated the health impact of diets comprised of UPFs. The most notable randomized controlled trial (RCT) to date involved 20 weight‐stable young adults who were admitted to the NIH Clinical Center and randomized to receive either ultra‐processed or unprocessed/minimally processed diets for 2 weeks immediately followed by the alternate diet for 2 weeks (Hall et al., 2019). Meals were designed to be matched for calories, energy density, macronutrients, sugar, sodium, and fiber. Participants consumed food ad libitum. Caloric intake was greater during the UPF diet (508 ± 106 kcal/day; *p* = 0.0001), and weight changes were highly correlated with energy intake (*r* = 0.8, *p* < 0.0001), with participants gaining 0.9 ± 0.3 kg (*p* = 0.009) during the UPF diet and losing 0.9 ± 0.3 kg (*p* = 0.007) during the UPF diet.

Despite the importance of the findings, this trial has been criticized on numerous grounds, including the short duration and the approximate twofold greater caloric density of the foods in the UPF diet. The diets were also not matched for types of fiber. It has also been suggested that the caloric intake in response to the two different diets would have converged with time. Meal eating rate was significantly greater during the ultra‐processed diet whether expressed as kcal/min or g/min, metrics that were moderately correlated with overall energy intake differences. The greater nonbeverage energy density and greater proportion of hyper‐palatable foods (using a previously proposed definition of the term (Fazzino et al., 2019) in the UPF diet each explained >40% of the meal energy intake differences between the high UPF and unprocessed/minimally processed dietary patterns (O'Connor et al., 2023) (Fazzino et al., 2023).

Energy density is largely determined by the water, fat, and fiber content and hyper‐palatability by various combinations of sodium, carbohydrate, and fat (Fazzino et al., 2019). Based on their definition of hyper‐palatability, Fazzino et al. (2019) determined that 62% (4795/7757) of foods in the Food and Nutrient Database for Dietary Studies meet the hyper‐palatable food criteria. Demeke et al. (2023) found that the prevalence of hyper‐palatable foods in the United States increased by 20% from 1988 to 2018 (49%–69%; *p* < 0.0001). According to these authors, it is likely that existing food products in the food system have been reformulated over time to enhance their palatability (Demeke et al., 2023). There is little doubt that food scientists better understand the flavors and tastes that increase food enjoyment and possibly consumption (Vasilaki et al., 2022).

Similar results to those of Hall et al. (2019) were recently reported by Hamano et al. (2024), who conducted an open‐label cross‐over randomized controlled trial (RCT) involving overweight/obese Japanese males randomly assigned (1:1) to a diet comprising either UPFs or non‐UPFs for 1 week, followed by a 2‐week washout period, before crossing over to the alternate food diet for 1 week. The meals were designed to be matched for total energy and macronutrient levels. During the UPF period, participants gained 1.1 kg more weight and consumed over 800 kcal more per day compared with the non‐UPF period. There appeared to be little difference in food enjoyment between the two diets based on visual analog scales used to assess a variety of relevant metrics. With respect to potential mechanisms, the number of chews per calorie was significantly lower during the UPF period. It has been hypothesized that food processing techniques yield a softer texture, which reduces the masticatory effort required to eat them (Lieberman et al., 2004; Zink & Lieberman, 2016). Moreover, a previous clinical trial showed a negative correlation between the number of chews per calorie and both total energy intake and waist circumference (Zink & Lieberman, 2016).

## 5 PROPOSED MECHANISMS FOR THE EFFECTS OF UPFS

In addition to energy density and hyper‐palatability, disruption of the food matrix has been proposed as being central to the concept of UPFs (Fardet, 2024). Fardet and Rock (2022) speculate that the worldwide chronic disease epidemic is mostly associated with the degradation and artificialization of food matrices rather than food composition. Artificialization is defined as the addition of cosmetic agents (coloring and texturizing agents, taste enhancers, aromas, etc.) to food or the creation of unnatural food matrices from purified isolated ingredients. Many possible mechanisms for the adverse health effects noted in observational studies have been proposed, although, at least with respect to obesity, one of the main health outcomes linked with UPF intake, none have robust support (Valicente et al., 2023).

As recently highlighted in an editorial in Lancet Gastroenterol Hepatol (The Lancet Gastroenterology Hepatology, 2025), the robust evidence required for policy and/or regulatory action on UPFs does not exist. It has not been shown that there is something inherent to all UPFs, such as PBMAs, that would make them unhealthful. In particular, and as highlighted below, there is a lack of identified unique biological mechanisms to explain the associations between UPF intake and adverse health outcomes.

In 2023, Valicente et al. (2023) grouped the hypothesized mechanisms for the effects of UPFs on body weight into three categories: food composition, food choice (namely low cost, shelf‐life, food packaging, hyper‐palatability, and stimulation of hunger/suppression of fullness), and digestive processes (namely oral processing/eating rate, gastric emptying time, gastrointestinal transit time, and microbiome). Based on their review, these authors concluded that there is “ … no mechanistic evidence directly linking UPF intake with increased body mass index …” and that as a result, adoption of the Nova system for dietary guidance is not justified at this time (Valicente et al., 2023).

As noted, faster eating rate, when expressed as g/min and especially kcal/min, is likely a factor contributing to the overconsumption of UPFs. In their cross‐over study involving 50 healthy‐weight participants who consumed four ad libitum lunch meals consisting of “soft minimally processed,” “hard minimally processed,” “soft ultra‐processed,” and “hard ultra‐processed” components, Teo et al. (2022) concluded that texture‐based differences and meal energy density contribute to observed differences in energy intake between minimally processed and ultra‐processed meals. In alignment with these findings are those from Fazzino et al. (2023), who found that across 2733 meals from four dietary patterns, ad libitum meal energy intake is positively influenced by energy density, eating rate, and hyper‐palatability.

Finally, it has been argued that UPFs are addictive. Using standards used to establish addictive nature of tobacco, Gearhardt and DiFeliceantonio (2023) concluded that highly processed foods can be categorized similarly. These standards are (1) cause highly controlled or compulsive use, (2) cause psychoactive (i.e., mood‐altering) effects via their effect on the brain, and (3) reinforce behavior and trigger strong urges or craving. According to LaFata et al. (2024), 14% of adults and 15% of children globally are addicted to these foods. However, although the idea that UPFs are addictive is an intriguing concept, it remains speculative. Hebebrand and Gearhardt (2021) highlighted at least five reasons why transference of the diagnostic criteria for substance use disorders to define food addiction is too simplistic.

## 6 DIVERGENT EFFECTS OF UPF SUBCATEGORIES IN OBSERVATIONAL STUDIES

Although epidemiologic findings consistently show associations between total UPF intake and adverse health outcomes, the evidence suggests that these associations are driven by just a handful of foods. Recently published observational studies have shown that many subgroups of UPFs are either not associated with risk or are associated with a decreased risk. Two recent analyses of three large US cohorts, the Nurses’ Health Study (NHS), NHS II, and the Health Professional Follow‐Up Study, illustrate this point (Chen et al., 2023). In one analysis, among the nearly 200,000 men and women who were followed for 24–30 years, 19,503 cases of type 2 diabetes (T2D) were identified. The adjusted hazard ratio (HR) for T2D comparing extreme quintiles of total UPF intake (percentage of grams per day) was 1.46 (95% confidence interval [CI] 1.39, 1.54). However, the HRs for 7 of the 14 subcategories of UPF were below 1.00, indicating a protective effect, and for 7 it was above 1.00, indicating an increased risk. In the other analysis, during the approximate 30‐year follow‐up, there were nearly 17,000 cases of cardiovascular disease (CVD) (Mendoza et al., 2024). The adjusted HR (95% CI) for CVD when comparing the highest versus lowest total UPF intake quintile was 1.11 (1.06, 1.16). However, for 7 of the 13 subcategories of UPFs, the HR was below 1.00. Notably, the association between total UPF and CVD was driven primarily by two subcategories: sugar‐sweetened beverages (HR, 1.19) and processed red meat, poultry, and fish (HR, 1.21).

Two additional analyses are especially relevant (Dicken et al., 2024; Li et al., 2024). In the UK Biobank study, which involved almost 65,000 men and women, total UPF intake was associated with shorter leukocyte telomere length (LTL) (Li et al., 2024). Aging is characterized by a progressive shortening of telomeres, which compromises their structure and function (Gruber et al., 2021). Again, however, there were divergent effects of the different subcategories of UPFs. Especially notable is that animal‐based UPFs (eggs in sandwiches, sausage, crumbed or deep‐fried poultry, bacon, ham, breaded fish, and battered fish) were associated with a shorter LTL, whereas vegetarian alternative UPFs (vegetarian sausages/burgers, Quorn, and other vegetarian alternatives) were associated with longer LTL. Similarly, a prospective cohort analysis of the European Prospective Investigation into Cancer and Nutrition involving over 300,000 adults showed that total UPF intake was associated with a marked increased risk of developing T2D (Dicken et al., 2024). During the nearly 11‐year follow‐up period, more than 14,000 individuals developed this disease. However, although total UPF was associated with an increased risk, there were divergent associations among the UPF subcategories. Most notable is that plant‐based meat and dairy alternatives, which were classified as UPFs, were associated with a more than 50% decreased risk, whereas intake of animal‐based UPFs, which consist of processed meat (beef, pork, and fish) and processed cheese, was associated with a more than twofold increased risk.

Finally, in a perspective by Visioli et al. (2024), the authors suggest this divergence indicates that there is a high possibility that the associations between processing and adverse health outcomes do not have a causal basis and are likely due to some unidentified lifestyle differences, while still allowing for the possibility that some aspects of processing/formulation, such as the use of certain emulsifies or additives could exert adverse effects. Regardless, the epidemiologic data indicate that some UPFs positively contribute to health.

## 7 HEALTH IMPACT OF SOY‐ AND NON‐SOY‐BASED MEAT AND DAIRY ALTERNATIVES

Despite their designation as UPFs, Messina et al. (2022) concluded after evaluating the existing literature that neither soymilk nor soy burgers exhibit the common attributes of UPFs (low satiety, high caloric density, fast eating rate, hyper‐palatability, etc.) more so than cow's milk and ground beef, which are classified as Group 1 foods. Furthermore, plant‐based meats and milk have been found to exert health benefits in comparison to animal‐based counterparts. One example comes from a systematic review and meta‐analysis of RCTs comparing the health effects of cow's milk with soymilk (Erlich et al., 2024). In the 17 trials that met the eligibility criteria (*n* = 504 adults with a range of health statuses), in which 19 CVD intermediate outcomes were assessed, the median daily dose of soymilk (22 g soy protein and 17.2 g or 6.9 g/250 mL added sugars) and cow's milk (24 g milk protein and 24 g or 12 g/250 mL total sugars as lactose) was 500 mL. The substitution of soymilk for cow's milk resulted in statistically significant reductions in low‐density lipoprotein‐cholesterol (LDL‐C) (0.19 mmol/L, ∼6%), systolic blood pressure (8.00 mmHg), diastolic blood pressure (4.74 mmHg), and C‐reactive protein (−0.82 mg/L, ∼22%) and very modest increases in high‐density lipoprotein‐cholesterol (HDL‐C) (0.05 mmol/L, 4%). No other outcomes showed differences, and there was no meaningful effect modification by added sugars across outcomes.

This analysis provides a strong counter‐argument to the assumption that UPFs are necessarily unhealthful since soymilk is classified as an UPF (Group 4), whereas cow's milk is Nova‐classified as a Group 1 food (unprocessed/minimally processed). It is also notable that soy protein isolate and soy protein concentrate have been shown to directly lower circulating LDL‐C concentrations, even though these concentrated sources of protein, and any food containing them, are classified as ultra‐processed (Blanco Mejia et al., 2019; Jenkins et al., 2019).

Three RCTs that evaluated the health effects of PBMAs are especially relevant to a discussion of Nova. In the Study With Appetizing Plantfood‐Meat Eating Alternative Trial (SWAP‐MEAT) (Crimarco et al., 2020), participants (*n* = 36) consumed about 2.5 servings daily of PBMAs or analogous meat products, providing 25% of total calories and 50% of total protein, for 8 weeks each in a randomized, crossover design. Consumption of PBMAs significantly decreased body weight and circulating levels of trimethylamine oxide (TMAO) and LDL‐C, when compared with the meat products (Crimarco et al., 2020). There were no differences in inflammatory markers between the groups nor were adverse effects observed on any of the other endpoints analyzed (Crimarco et al., 2022). In the SWAP‐MEAT Athlete study, 24 athletes (12 recreational runners and 12 resistance trainers) were randomly assigned to three diets for 4 weeks each in a crossover design: whole foods plant‐based, plant‐based with PBMAs, and an omnivorous diet (Roberts et al., 2022). At study termination, there were no differences in running outcomes for the runners or lifting outcomes for the resistance trainers, indicating that inclusion of PBMAs in a plant‐predominant diet may not impact fitness outcomes in athletes compared to a whole foods plant‐predominant diet and an omnivorous diet.

Finally, there is a similarly designed trial by Toh et al. (2024) but that utilized a parallel design and intervened with PBMAs mostly comprised of soy protein rather than pea protein as in the SWAP‐MEAT (Crimarco et al., 2020). In contrast to the findings from the SWAP‐MEAT (Crimarco et al., 2020), no differences between groups were noted in effects on LDL‐C and TMAO. Moreover, although homeostatic model assessment for beta‐cell function improved in both groups, there was no difference between groups. Diastolic blood pressure decreased in response to the PBMAs, whereas glycemic homeostasis was better regulated in the control group. The differing macronutrient/fiber content of the PBMAs in the trials by Crimarco et al. (2020) and Toh et al. (2024) likely explain the different results. Energy and saturated fat intake by the plant‐based group was lower than in the control group in the trial by Crimarco et al. (2020) but not in the trial by Toh et al. (2024).

## 8 UPFS IN PLANT‐BASED DIETS

According to Hallinan et al. (2021), some UPFs are needed for nutrient‐adequate diets. In the case of plant‐based diets, foods that have been classified as UPFs, including plant‐based milks, and meat analogs, have value as sources of critical nutrients for both vegetarians and those who eat traditional plant‐based diets. There is, however, a wide variation in nutrient content of plant‐based milks and meats. For example, Drewnowski (2021) determined that 90% of US plant‐based milk alternatives are Nova‐classified as UPFs. Of the 641 plant milks analyzed in a separate undertaking, about one third were not fortified with calcium at a level similar to cow's milk, only 20% contained similar amounts of protein, and only about 5% matched the energy, sugar, calcium, protein, and vitamins D, A, and B12 content (Drewnowski et al., 2021).

Similarly, an analysis of 28 PBMAs by Cole et al. (2022) showed large differences in calorie, nutrient, and fiber content per 100 g product as illustrated by the mean values and standard deviations. For example, values for calories, fiber (g), total fat (g), and saturated fat (g) were 193.60 ± 71.93, 4.66 ± 2.63, 8.72 ± 6.59, and 2.14 ± 2.46, respectively. There were also large differences in mineral content. In contrast, most burgers contained large amounts of protein (g), and the differences among types were less marked (19.83 ± 5.97) (Cole et al., 2022). The variation in the nutrient composition of plant milks and PBMAs raises questions about the relevance of Nova for evaluating the health impacts of these products as it is unlikely that a calcium‐ and vitamin D‐fortified plant milk, containing 7–8 g protein per 250 mL, will have similar impacts on health as unfortified plant milk containing 1 g protein. It is imperative that consumers understand that the macro‐ and micronutrient contents of plant‐based meat and milk alternatives vary significantly.

One potential advantage of the new generation of PBMAs is that these products can be fortified with shortfall nutrients in plant‐based diets, such as zinc, iron, and vitamin B12. However, little is known about the bioavailability of micronutrients from these products. Although many PBMAs are higher in sodium than meat (Alessandrini et al., 2021; Liaw et al., 2023; Yeo et al., 2023), these comparisons typically fail to consider the salt added during meat preparation. It is likely that future iterations of PBMAs will be lower in sodium to be better aligned with government guidelines calling for reduced sodium content in foods.

Plant‐based meats based on isolated plant proteins can make valuable contributions to the diets of those who are at risk of not getting adequate protein, such as the elderly and possibly vegans. A recent simulation study by Borkent et al. (2024) involving older Dutch consumers revealed that in comparison to the standard Dutch diet, utilizable protein intake decreased by about 5% in the flexitarian, pescetarian, and vegetarian scenarios, whereas in the vegan scenario, both total protein intake and utilizable protein were lower, leading to nearly 50% less utilizable protein compared to the standard Dutch diet. PBMAs may be particularly helpful in meeting protein needs because many versions are higher in protein on a caloric basis than dry beans (Messina et al., 2022), and dry beans can cause gastrointestinal disturbances in some individuals (Widham & Hutchins, 2011). Based on their cross‐sectional analysis, Brazilian researchers recently concluded that UPFs such as PBMAs, in vegan diets are common protein sources that can help avoid nutrient deficiencies and may not be associated with the detrimental health outcomes linked with other types of UPFs (Leitao et al., 2024).

Although protein content is similar between the new generation of PBMAs and the products they are intended to replace, protein quality must also be considered. In general, based on indispensable amino acid content and digestibility, animal protein is of higher quality than plant protein. Only limited research has evaluated the quality of protein when in the form of a PBMA, whereas a considerable amount of work has evaluated the quality of concentrated forms of soy protein and to a lesser extent pea protein, two of the main proteins used in meat alternatives. Soy protein is a high‐quality protein (USDA, 2022) based on the results of nitrogen balance studies (Young, 1991) and as determined by the protein digestibility amino acid score, the method used by most regulatory bodies for evaluating protein quality (Hughes et al., 2011; Rutherfurd et al., 2015) and the digestible indispensable amino acid score (Rutherfurd et al., 2015), a newer method widely discussed in the literature but not adopted by regulatory agencies. Pea protein is a lower quality protein than soy protein but is still a good quality protein (Rutherfurd et al., 2015). It is important to consider the quality of protein in the context in which it is consumed because of the possible effects of the food matrix and processing. Limited data indicate that the quality of protein in the form of a soy burger (Impossible Burger) is comparable to the quality of beef protein, whereas the quality of the protein in a pea protein‐based burger (Beyond Burger) is about 20% lower than beef and soy protein (Fanelli et al., 2022).

For at least two reasons, in high‐income countries, protein quality may not be an issue relevant to the consumption of PBMAs. First, the overall protein quality of the diet should be the focus because proteins with complimentary amino acid profiles can combine to form higher quality proteins (Dimina et al., 2021). Second, consuming, for example, 20 g/day of a somewhat lower quality protein in place of a somewhat higher quality protein is unlikely to impact protein nutriture in high‐income populations given that the protein intake of most people greatly exceeds the recommended dietary allowance, although there may be some exceptions (Moughan et al., 2024).

Finally, PBMAs can help consumers transition to a plant‐forward diet with a smaller carbon footprint despite the detrimental environmental effects of UPFs in general. According to Leite et al. (2022), “ultra‐processed food production … uses large quantities of land, water, energy, herbicides and fertilisers; and causes eutrophication and environmental degradation from greenhouse gas emissions and accumulation of packaging waste as well as species loss, all this is liable to cause ecosystem collapse, further affecting biodiversity.” However, even with the processing involved in the manufacture of plant milks and plant meats, these products have lower environmental impacts than their animal‐based counterparts (Coffey et al., 2023). According to a comprehensive analysis by Springman, processed plant‐based foods such as veggie burgers and plant milks were [found to be] associated with less climate benefits and greater costs than unprocessed foods but still offered substantial environmental, health, and nutritional benefits compared to animal‐source foods (Springmann, 2024).

## 9 SUMMARY AND CONCLUSIONS

The development of Nova has served to focus attention on the potential impact of processing/formulation on health effects of foods independent of nutrient content. Several trials assessing the health effects of diets high in UPFs are underway or planned. Nova has also served to highlight the value of basing diets on unprocessed/minimally processed foods although this message is not novel. Nevertheless, we believe that nutrient content should be the primary metric by which the healthfulness of foods is determined.

Several lines of evidence suggest that the Nova food classification system lacks sufficient nuance to guide food purchasing decisions and to dictate public health guidelines. Nova demonizes some nutrient‐dense foods which might be classified as UPFs simply because they contain an emulsifier or food additive which may or may not affect health. Nova also potentially distracts consumers from focusing on the importance of nutrient content.

Based on the results of clinical research and epidemiological studies, certain plant foods, including plant milks and PBMAs, do not lead to and are not associated with adverse health effects unlike other UPFs, including ultra‐processed animal foods. These plant foods may also contribute critical nutrients to plant‐based diets. Classifying a fruit smoothie made with two Group 1 foods (fruit and cow's milk) as an UPF if it contains a concentrated source of protein can discourage consumers from preparing and consuming foods needed to meet their nutrient needs and also serves to solidify the negative perception of any type of food processing in the minds of the public. Nevertheless, more short‐ and long‐term clinical trials evaluating plant‐based meat and dairy alternatives are warranted, the former looking at metrics such as nutrient bioavailability, and the longer looking at clinically relevant markers of health.

## AUTHOR CONTRIBUTIONS

**Mark Messina**: Conceptualization; writing—original draft; writing—review and editing. **Virgina Messina**: Writing—review and editing.

## CONFLICT OF INTEREST STATEMENT

Mark Messina is the Director of Nutrition Science and Research for Soy Nutrition Institute Global, which receives funding from the United Soybean Board. Virgina Messina has no conflicts to report.
